# Intracranial Angioplasty Utilizing the Comaneci Device As Bailout in Very Late Thrombectomy for Ischemic Stroke

**DOI:** 10.7759/cureus.88616

**Published:** 2025-07-23

**Authors:** Diego A Ortega-Moreno, Adam A Dmytriw, Adrián Peña-Fuentes, Fernando Góngora-Rivera

**Affiliations:** 1 Department of Neurology, Hospital Universitario "Dr. Jose Eleuterio Gonzalez", Monterrey, MEX; 2 Interventional Neuroradiology, St. Michael's Hospital, Toronto, CAN; 3 Radiology, Harvard Medical School, Boston, USA; 4 Neurology and Neurosurgery Institute, Zambrano Hellion Medical Center, Monterrey, MEX

**Keywords:** brain thrombectomy, comaneci device, endovascular angioplasty, intracranial atherosclerotic disease, ischemic stroke

## Abstract

Endovascular management of ischemic stroke has been established as an effective treatment option. Nevertheless, restrictive selection criteria and limited device availability may delay recanalization in certain cases, particularly in developing countries. This report describes a resource-driven, off-label use of the Comaneci device in a patient with ischemic stroke presenting beyond the 24-hour window. A 61-year-old male arrived 28 hours after last known well (LKW). Despite initial clinical improvement with medical therapy alone, neurological deterioration occurred 2 days after onset. Primary aspiration thrombectomy achieved incomplete recanalization (mTICI 1, antegrade reperfusion but limited distal branch filling), revealing underlying intracranial atherosclerosis. Due to a lack of standard angioplasty tools, mechanical angioplasty with a Comaneci petit device was performed, improving perfusion to mTICI 2c (near complete perfusion except for slow flow or distal emboli). At 3-month follow-up, the patient had no residual symptoms and achieved full recovery (National Institutes of Health Stroke Scale (NIHSS) 0, mRS 0). Endovascular management remains a critical component of stroke care. Further exploration of alternative and adaptable strategies may help expand treatment access in challenging clinical settings.

## Introduction

Mechanical thrombectomy (MT) has been established as an effective treatment for acute ischemic stroke (AIS), either following or independent of intravenous thrombolysis, in appropriately selected patients based on clinical and imaging criteria within a defined time window [1]. Typically, MT is restricted to within 24 hours of symptom onset due to evidence from landmark trials demonstrating the greatest benefit and safety within this period. Beyond 24 hours, the role of MT remains uncertain, and guidelines provide limited recommendations, creating a clinical gap for delayed interventions.

Intracranial atherosclerotic disease (ICAD) is an important cause of AIS, characterized by stenosis of intracranial arteries, which can complicate mechanical reperfusion strategies and contribute to recurrent or progressive ischemia [2]. Adjuvant revascularization techniques, including balloon angioplasty and intracranial stenting, are frequently employed when thrombectomy fails or reveals underlying ICAD. However, in resource-limited settings, access to conventional angioplasty tools may be restricted.

We report a unique case of delayed MT performed beyond the 24-hour window, followed by off-label angioplasty using a Comaneci petit device (Rapid Medical, Yokneam, Israel), a device approved by the FDA for temporary assistance during coil embolization of wide-necked aneurysms. To our knowledge, this is the first documented use of the Comaneci device for mechanical angioplasty in the setting of ICAD during acute stroke management, addressing a clinical need where conventional balloon angioplasty was unavailable.

## Case presentation

A 61-year-old male with a history of hypertension and tobacco use experienced a transient ischemic attack characterized by right hemiparesis and motor aphasia lasting two hours. The following morning, he awoke with recurrent symptoms suggestive of a new ischemic insult and arrived at the emergency department 28 hours after last known well (LKW) with an NIHSS score of 12. Due to the delayed presentation after symptom onset, he was admitted for supportive medical management, including glycemic and blood pressure optimization. Non-contrast computed tomography (CT) revealed an established ischemic lesion in the posterior limb of the internal capsule in the left cerebral hemisphere.

**Figure 1:**
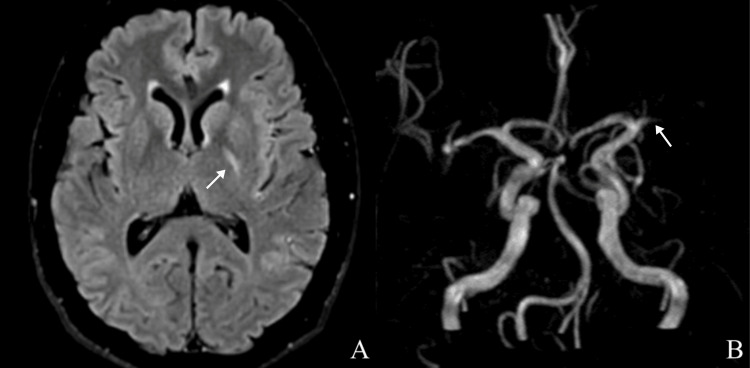
A) T2-FLAIR MRI demonstrating a hyperintense lesion localized in the posterior limb of the left internal capsule (arrow indicates lesion site). B) Angio-MRI with a 3D reconstruction of the cerebral vasculature, demonstrating a vascular occlusion at the left middle cerebral artery (arrow points to the site of neurovascular occlusion).

The patient initially showed gradual partial neurological improvement under continued medical management, reaching an NIHSS score of 6 at 40 hours from symptom onset. However, at 44 hours, he experienced clinical deterioration with new findings of dense right hemiparesis, hypoesthesia, and global aphasia, resulting in an NIHSS score of 18. Magnetic resonance imaging (MRI) of the brain parenchyma demonstrated a left middle cerebral artery (MCA) occlusion, located at the M1 segment (Figures 1A-1B).

No perfusion imaging was performed on CT or MRI due to unavailability; nevertheless, given the patient’s significant clinical deterioration and the absence of clear consensus in current guidelines regarding thrombectomy beyond 24 hours from stroke onset, cerebral angiography was performed to evaluate collateral circulation to the hypoperfused region and assess the feasibility of endovascular intervention.

**Figure 2:**
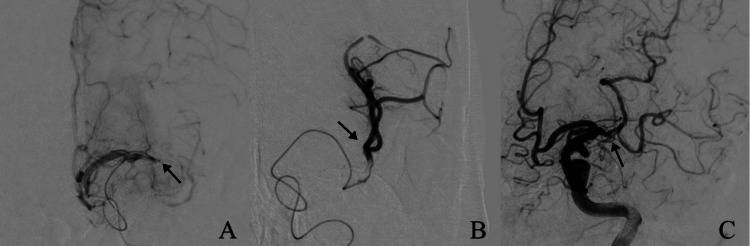
A) Cerebral angiography demonstrating the absence of distal vascular flow in the left middle cerebral artery territory (arrow points to the site of neurovascular occlusion). B) Selected distal middle cerebral artery angiography with microcatheter demonstrating distal arterial permeability (arrow indicates microcatheter tip beyond the occlusion site, injecting contrast). C) Digital subtraction angiography demonstrating partial recanalization after attempted first-pass aspiration thrombectomy (arrow shows site of residual stenosis consistent with underlying ICAD). ICAD: Intracranial atherosclerotic disease

A cerebral angiogram demonstrated an arterial occlusion at the M1 segment of the left MCA (Figure 2A). A Benchmark catheter (Penumbra; California, USA) was placed in the internal carotid artery, followed by a PX SLIM 0.025 microcatheter (Penumbra; California, USA) with a 0.014 guidewire. Distal MCA angiography demonstrated post-occlusive arterial patency (Figure 2B). Initial primary aspiration with a Penumbra system resulted in partial recanalization with severe residual stenosis, classified as grade 1 on the modified Treatment In Cerebral Infarction scale (mTICI 1; antegrade reperfusion but limited distal branch filling), due to simultaneous underlying ICAD (Figure 2C).

**Figure 3:**
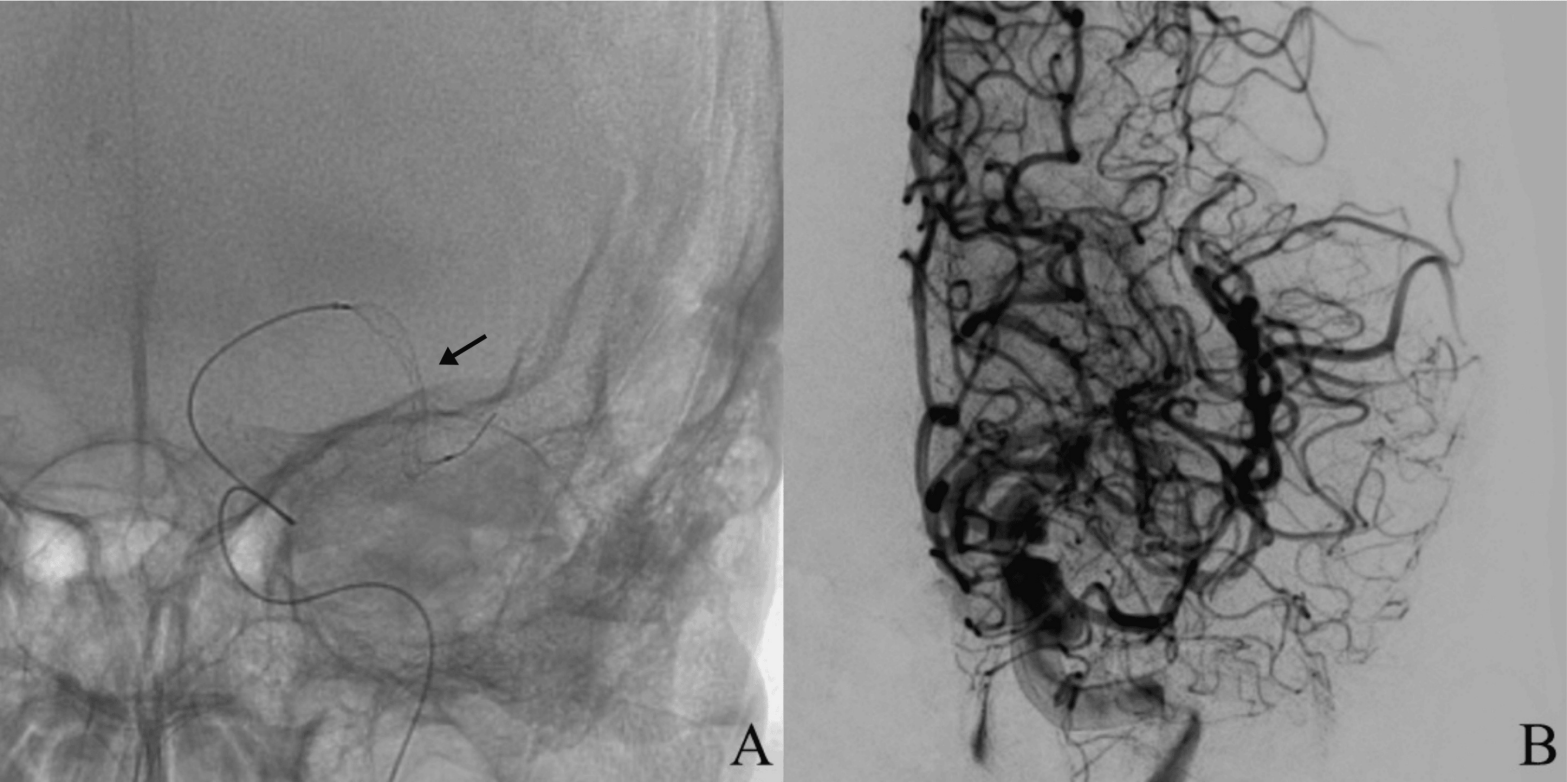
A) A Comaneci petit device being deployed at the occlusion site as an angioplasty mechanism (arrow marks Comaneci petit device location and deployment). B) Cerebral angiography after endovascular intervention demonstrating restoration of vascular flow in the left middle cerebral artery territory.

After identification of partial recanalization due to ICAD, adjuvant angioplasty was considered the most adequate management option. However, at this center, the lack of conventional intracranial angioplasty tools was a limiting factor. After an in-depth discussion, considering the clinical and mechanical characteristics of the Comaneci device (Rapid Medical, Israel), it was decided to utilize a Comaneci petit size device. Angioplasty was performed with three diameter-progressive dilations until partial recanalization was achieved (mTICI 2c) with 30% residual arterial stenosis (Figures 3A-3B).

After the procedure, dual antiplatelet therapy (using clopidogrel and aspirin) was initiated for 21 days. Twenty-four hours after the endovascular intervention, the patient´s condition further improved, reporting an NIHSS score of 4. Follow-up after 3 months demonstrated no residual motor, sensorial, or language symptoms (modified Rankin scale score of 0).

## Discussion

To our knowledge, this is the first reported use of the Comaneci device for mechanical angioplasty in the setting of ischemic stroke with underlying ICAD beyond the 24-hour window. MT with stent-retriever or primary aspiration has been established as the preferred mechanism for reperfusion acquisition in AIS, due to its recanalization rates and number of associated complications, particularly in proximal vessel occlusion of the anterior circulation, small infarct core, and presence of adequate collateral circulation [3]. However, several studies and meta-analyses have shown that patients with larger infarct cores, as well as distal arterial occlusion, may still benefit from MT [4]. Current stroke knowledge indicates that mechanical reperfusion within a 24-hour onset is a secure practice [5,6]. Nevertheless, the 24-hour window implies that only a limited proportion of patients with stroke are eligible for mechanical reperfusion.

Consequently, continuous global efforts are being made to establish the feasibility and safety of delayed mechanical reperfusion (>24 hrs.), especially in patients with progressive clinical presentations and ischemic claudication associated with intracranial stenosis and acute vascular occlusion. Currently, various clinical studies indicate that delayed endovascular intervention may benefit patients’ clinical presentation, but insufficient reliable studies on combined delayed mechanical reperfusion burden decision-making for endovascular treatment beyond 24 hours after a time known well [7,8]. A limitation in this case was the absence of perfusion imaging performance due to the unavailability of this imaging modality at our institution, as is the case in many resource-limited settings. However, the decision to proceed endovascularly was based on significant clinical worsening, radiographic evidence of occlusion, and existing literature supporting the potential benefit of extended-window thrombectomy in selected patients.

Similarly, angioplasty has been evaluated and established as a feasible rescue treatment modality after failed primary MT [9,10]. The number of mechanisms and techniques for mechanical reperfusion is under continuous development and innovation. However, their general objective remains similar: to achieve adequate reperfusion within a smaller time frame and with fewer associated complications. The Comaneci Embolization Assist Device is an FDA-approved temporary device designed to facilitate coil embolization of wide-necked intracranial aneurysms (neck width 4-10 mm). It consists of 12 radiopaque nitinol wires forming a mesh at the distal end, mounted on a flexible shaft, allowing an operator-dependent expansion and contraction, controlled through a handle with a slider [11]. Though primarily designed to assist coil embolization of intracranial aneurysms, the Comaneci device functions similarly to developed angioplasty mechanisms. Its radiopaque distal wire mesh enables precise localization under fluoroscopy during endovascular intervention. This design allows satisfactory operator-controlled expansion, facilitating evaluation of reperfusion acquisition and residual stenosis after angioplasty attempt.

The Comaneci device provides an acceptable radial force when expanded, increasing consecutively with each diameter dilation. The previously described mechanical and functional characteristics of the Comaneci device are comparable to those of other endovascular techniques used for mechanical reperfusion acquisition. For instance, the 6-mm Solitaire stent-retriever, when deployed within a 1.5 mm vessel, can exert a radial force of 0.02 N/mm, decreasing to 0.015 N/mm in a 3 mm vessel. In comparison, the Comaneci 17 can apply a radial force of 0.06 N/mm in a 1-millimeter vessel, decreasing to approximately 0.04 N/mm in a 3 mm vessel [12]. The characteristics of this device facilitate reperfusion acquisition on a previously stenosed vessel without the transient arterial occlusion associated with other angioplasty devices, while permitting intraoperative evaluation of the dilation extent [13].

The use of the Comaneci device for angioplasty is currently off-label, although various initial clinical attempts have been documented evaluating its feasibility and safety in alternative endovascular treatment modalities, particularly as an angioplasty mechanism for vasospasm after subarachnoid hemorrhage, demonstrating its safety and functionality [14-19]. In our late-onset stroke case, the Comaneci device proved to be an effective and safe angioplasty device. The decision to use the Comaneci device in this case was driven by several patient- and context-specific factors. The patient showed significant neurological worsening (NIHSS increased from 6 to 18), and imaging revealed persistent vessel occlusion despite aspiration thrombectomy, with residual stenosis suggestive of underlying ICAD. Due to the absence of balloon angioplasty tools in our resource-limited setting, and favorable anatomy for device navigation and expansion, the Comaneci device was selected as an off-label alternative. This intervention led to improved cerebral perfusion and full neurological recovery, reinforcing the feasibility of rescue angioplasty beyond conventional time windows when guided by clinical deterioration and vascular imaging. These findings support the need to evaluate similar non-occlusive devices for angioplasty in ICAD-related stroke.

This case report has several limitations that should be acknowledged. First, as a single case, the findings cannot be generalized, and prospective studies are needed to confirm the safety and efficacy of using the Comaneci device for angioplasty in ICAD, vasospasm, and other vaso-occlusive scenarios. Second, the absence of advanced perfusion imaging limited our ability to more precisely assess the ischemic penumbra and guide intervention, a constraint common in many resource-limited settings. Third, the off-label use of the Comaneci device, while promising in this case, requires further evaluation to understand its comparative effectiveness and potential risks relative to standard angioplasty techniques. Last, the generalizability of our findings is limited by both the single-case design and the lack of confounding assessments such as perfusion imaging, collateral grading, and ischemic core quantification. Future research should focus on systematic evaluations of such devices in larger cohorts to better define indications, optimize patient selection, and establish standardized protocols for delayed endovascular intervention in AIS.

## Conclusions

This single case highlights that, in selected patients with ICAD and progressive neurological deterioration beyond the conventional 24-hour window, endovascular intervention may still be appropriate. Furthermore, in this context, the off-label use of the Comaneci petit device as an alternative angioplasty tool, prompted by the unavailability of standard balloon catheters, proved technically feasible and clinically effective, leading to substantial reperfusion and full neurological recovery without complications. These findings support the consideration of adaptable treatment strategies using available devices in resource-limited settings and warrant further evaluation in broader clinical contexts.
